# Supplementary material for: Exploring the achievements and forecasting of SDG 3 using machine learning algorithms: Bangladesh perspective
Source: PLoS One. 2025 Mar 4;20(3):e0314466. doi: 10.1371/journal.pone.0314466 (PMC11878945; doi:10.1371/journal.pone.0314466)
Supplement: Attachment — Submitted filename: Second Round Revision.docx [file pone.0314466.s002.docx]

**The authors have made a notable effort in addressing the required revisions. However, there are still several key areas that require further enhancement**

- The current description in the Material and Methods section still appears insufficiently detailed, particularly regarding data sourcing and model specifics. The section lacks essential information, such as: data source, sample size and model details. More comprehensive details are needed about the models employed in the study. Please check some published articles of good journals, to know how to write these.
- We can only decide a model is generalized or not based on the test data statistics. As it is mentioned that 25% of the data is used for validation set. Please provides the results of the error matrices, based on these values.
- Give the citation of the statement: These models, ARIMA, BRNN, and ENET, were chosen because they are well-suited for handling small sample sizes or limited data points.
- Top of Form
- Calculating RMAE isn’t standard practice mainly because the square root doesn’t provide additional insights or advantages over MAE in the context of understanding model performance. Additionally, taking the square root of an average of absolute values doesn’t conform to a meaningful statistical measurement.
- No need to write the details of error matrices in point numbers. Write those in a paragraph style. Please check some published articles of good journals.
- The discussion provides a comprehensive analysis of SDG 3 in the context of Bangladesh and offers valuable insights into the country’s progress and challenges. However, the section lacks a comparative analysis with previous studies. Comparisons to prior research would strengthen the manuscript.
- Please add the funding details data availability and conflict of interest statements.
