# Supplementary material for: Exploring the achievements and forecasting of SDG 3 using machine learning algorithms: Bangladesh perspective
Source: PLoS One. 2025 Mar 4;20(3):e0314466. doi: 10.1371/journal.pone.0314466 (PMC11878945; doi:10.1371/journal.pone.0314466)
Supplement: Attachment — Submitted filename: Response_to_Reviewers_auresp_4.docx [file pone.0314466.s006.docx]

Md. Maeen Molla

Lecturer

Department of Statistics

Bangabandhu Sheikh Mujibur Rahman Science and Technology University, Pirojpur

10 November 2024

PLOS ONE

**Rebuttal Letter for Manuscript [PONE-D-24-00392 and EMID:5f662cd9eff2f8bc]**

Dear sir,

We would like to extend our heartfelt appreciation to the academic editor and reviewers for generously devoting their time and offering insightful feedback on our manuscript titled "Exploring the achievements and forecasting of SDG 3 using machine learning algorithms: Bangladesh perspective." We are grateful for the constructive comments provided, as they have enabled us to enhance the overall quality and coherence of our research. In light of the raised concerns, we have meticulously addressed each point in a comprehensive manner as outlined below:

**Journal Requirements:** Please review your reference list to ensure that it is complete and correct. If you have cited papers that have been retracted, please include the rationale for doing so in the manuscript text, or remove these references and replace them with relevant current references. Any changes to the reference list should be mentioned in the rebuttal letter that accompanies your revised manuscript. If you need to cite a retracted article, indicate the article’s retracted status in the References list and also include a citation and full reference for the retraction notice.

**Reply:** We have thoroughly reviewed our reference list and can confirm that all citations are accurate and current. There are no retracted papers included in our references and we have not identified any need to replace or modify any entries.

**Additional Editor:** This very unfortunate that the comments given on the previous version of the manuscript has not been properly addressed like again I can see many smaller paragraphs throughout the manuscript. Authors are advised to read some good published papers on modelling.

**Reply:** We apologize for the misinterpretation of the previous comments. In this revision, we will make sure to properly address the issue, particularly by restructuring the manuscript to avoid the use of smaller paragraphs. We have read some good published papers on modelling published in reputed journals including PLOS ONE to address the issue.

**Additional Editor:** Following subheadings should be merged into one paragraph

AIC, R2, RMSE, RMAE in Materials and methods section into one paragraph.

Results section: Neuron, Lag, Model adaptation, Temporal consideration, Model complexity

Alpha (fraction), Lambda, Lag, Consideration for alpha (fraction) parameter, Consideration for lambda and regularization

Delete these subheads and make a running paragraph.

**Reply:** We have merged the mentioned subheadings into cohesive paragraphs as suggested. The “Materials and methods” section is revised accordingly and the “Results section” is streamlined by removing the subheadings to ensure a more fluid and readable narrative.

**Additional Editor:** L258-260: Should be part of ENET section while it is now under BRNN section.

**Reply:** We have moved lines 258-260 to the ENET section to ensure the information is correctly placed under the appropriate context.

**Additional Editor:** Alpha (fraction), Lambda, Lag, Consideration for alpha (fraction) parameter, Consideration for lambda and regularization

I can see paragraphs of 1-2 line length throughout the manuscript. Please try to combine smaller paragraphs into a paragraph of half a page length wherever possible.

**Reply:** We will revise the manuscript to combine the smaller paragraphs into longer, more cohesive ones, aiming for approximately half a page in length wherever appropriate. This will help improve the flow and readability of the text.

**Additional Editor:** Please improve the figure quality.

**Reply:** We have enhanced the quality of the figures to ensure they are clear and visually informative.

We've incorporated all feedback from the editor, ensuring the revised manuscript aligns with PLOS ONE's high standards. We do believe the changes substantially enhance the research quality and contribution. Additionally, we are interested to publish the whole reviewing process too.

We reiterate our deep appreciation for their invaluable expertise and insights that greatly improved our work. We trust this revised version merits publication in PLOS ONE.

Thank you for your time and consideration.

Sincerely,

On behalf of all the authors

Md. Maeen Molla (E-mail: [s1710624163@ru.ac.bd](mailto:s1710624163@ru.ac.bd))

Lecturer

Department of Statistics

Bangabandhu Sheikh Mujibur Rahman Science and Technology University, Pirojpur

Pirojpur-8500, Bangladesh
