# Supplementary material for: Exploring the achievements and forecasting of SDG 3 using machine learning algorithms: Bangladesh perspective
Source: PLoS One. 2025 Mar 4;20(3):e0314466. doi: 10.1371/journal.pone.0314466 (PMC11878945; doi:10.1371/journal.pone.0314466)
Supplement: Attachment — Submitted filename: Response to Reviewers.docx [file pone.0314466.s003.docx]

Md. Maeen Molla

M.Phil. Fellow

Department of Statistics

University of Rajshahi

Rajshahi, Bangladesh-6205

21 March 2024

PLOS ONE

Rebuttal Letter for Manuscript [PONE-D-24-00392 and EMID:5f662cd9eff2f8bc]

Dear Bappa Das,

We would like to extend our heartfelt appreciation to the academic editor and reviewers for generously devoting their time and offering insightful feedback on our manuscript titled "Exploring the achievements and forecasting of SDG 3 using machine learning algorithms: Bangladesh perspective." We are grateful for the constructive comments provided, as they have enabled us to enhance the overall quality and coherence of our research. In light of the raised concerns, we have meticulously addressed each point in a comprehensive manner as outlined below:

**Reviewer #1**: 1. Abstract: Abstract is written well but model performance statistics values are not incorporated. Add the statistics values of the model performance parameters to describe best performed model among the used models. In line 20 add the full form AIC as well.

Reply: Thank you sir for your valuable comment to enhance the quality of the abstract. We have added the statistics values of the model performance parameters to describe the best performed model as you mentioned and also added the full form AIC in line 20.

**Reviewer #1**: 2. Introduction: Introduction section is structured and written well by the authors. Add reference for the line 37-38 and 80-81.

**Reply:** We have given the appropriate references for the line 37-38 and 80-81.

**Reviewer #1**: 3. Materials and Methods: Materials and methods section of the manuscript is described well in detail, however the details of used statistic parameters such as R2, RMSE etc. are missing. The incorporation of few more important parameters such as Nash–Sutcliffe model efficiency coefficient is recommended. The 120th line of the results section can be described in materials and methods section rather than explaining it multiple times in results section of the manuscript.

**Reply:** In the method section, we have addressed the missing details of the statistical parameters, including $R^{2}$, RMSE, etc. The Nash-Sutcliffe Model Efficiency Coefficient (NSE) is a statistical measure that assesses the performance of hydrological or water quality models. However, we did not utilize this metric as it is specifically designed for simulated data and hydrological or water quality models. We have also moved the explanation of the 120th line to the materials and methods section to avoid repetition in the results section.

**Reviewer #1**: 4. Results: Results section of the manuscript is constructed and written well, incorporating the necessary findings of the study. Minor corrections are suggested in this section. The full form of used abbreviations should be provided at least once in the manuscript when it is firstly used such as ACF (in line 118), PACF (in line 116) etc. Line 120 and 137 can be transferred to the materials and methods section of the manuscript while discussing ARIMA model. In line 224 write ENET is recommended/better over the other two models rather than declaring the ENET as the best model.

**Reply:** We have made the necessary corrections based on your suggestions. We have provided the full form of abbreviations such as ACF and PACF when they were first used in the manuscript. Additionally, we have transferred the content from lines 120 and 137 to the materials and methods section where we discuss the ARIMA model. Lastly, we have revised line 224 to state that ENET is recommended/better over the other two models instead of declaring it as the best model.

**Reviewer #1**: 5. Conclusion: Conclusion is written well but the performances of the models’ used part is missing. The performances of the ML models used for this study should be addressed in this section with the future scope of the use of other ML methods for this purpose.

**Reply:** We have added a new table of the performance measures as table 6 and added “ENET generally outperforms BRNN and ARIMA, exhibiting lower RMAE and RMSE values for indicators such as NMR, U5MR, and Death Rate Due to RTI. For instance, in the case of NMR, ENET achieves an RMAE of 0.603446 and an RMSE of 0.451162, outperforming both BRNN and ARIMA. Similarly, for U5MR, MMR, and Death Rate Due to RTI, ENET consistently exhibits lower error metrics compared to the other models. Overall, the results suggest that ENET may offer superior predictive accuracy for these health indicators compared to BRNN and ARIMA models.” in the Conclusion section. Also added “In the future, there is great potential for using other ML methods to forecast health indicators and assess progress towards SDGs in Bangladesh. Deep learning models, gradient boosting models, time series forecasting with transformers, Bayesian methods, and ensemble methods are some ML approaches that can be explored. These methods offer opportunities to improve predictive accuracy, capture complex relationships, handle uncertainty, and enhance overall forecasting performance. Rigorous experimentation and evaluation, considering data availability, computational resources, and interpretability, will be crucial for selecting and implementing appropriate ML methods in this context.” as the response of the future scope of the use of other ML methods for this purpose.

Thank you sir, we are grateful for your valuable comments.

**Reviewer #2:** Introduction Section

To enhance the manuscript, the transition from a broad discussion on global SDG goals to a specific focus on Bangladesh’s achievements and challenges could be smoother and more interconnected. Incorporating references to prior research that specifically addresses Bangladesh's progress in meeting these goals would provide a more robust and contextual background. This approach would not only seamlessly connect global objectives with local realities but also underscore the study's relevance within the existing body of research.

**Reply:** To improve the transition from the broad discussion on global SDG goals to a specific focus on Bangladesh's achievements and challenges, we have restructured the content to create a smoother and more interconnected flow. We have incorporated references to prior research that specifically addresses Bangladesh's progress in meeting these goals. This addition provides a robust and contextual background, seamlessly connecting global objectives with local realities.

**Reviewer #2:** The introduction efficiently introduces the key health indicators (neonatal, under-5, and maternal mortality, and deaths from road traffic injuries). However, it could be enhanced by briefly discussing why these particular indicators are critical for Bangladesh.

**Reply:** We have added “To ensure the achievement of Sustainable Development Goals in Bangladesh, a specific set of four indicators has been chosen under SDG 3. These indicators include NMR, U5MR, MMR, and Death Rate Due to RTI. The selection of these indicators has been done under the guidance of the SDG Working Committee of The Prime Minister's Office. All relevant ministries are actively involved in this process. It would be helpful to understand why these indicators are critical for Bangladesh. These indicators are important because they reflect the overall health and well-being of the population, especially vulnerable groups like newborns, children under 5, and mothers. High neonatal, under-5, and maternal mortality rates indicate challenges in healthcare access, quality, and maternal health. Deaths from road traffic injuries highlight the need for improved road safety measures. By focusing on these indicators, Bangladesh can prioritize interventions and policies to improve healthcare and save lives.” in the Introduction section.

**Reviewer #2:** In the introduction mentions a brief explanation of why these particular methods were chosen and their relevance to the data at hand would be beneficial.

**Reply:** We have added “These models, ARIMA, BRNN, and ENET, were chosen because they are well-suited for handling small sample sizes or limited data points. ARIMA is specifically designed for forecasting equally spaced univariate time series data. BRNN, with its bidirectional structure, can effectively utilize information from both past and future states, which can be advantageous when working with a small sample size. ENET, as a regularization technique, can handle the multicollinearity problem and perform feature selection simultaneously, making it a good choice for models with limited data points.” in the Introduction section.

**Reviewer #2:** Additionally, it was mentioned in the text that “Previous studies have found that ARIMA modelling performed well in predicting Neonatal Mortality Rate (NMR) 81 and Under-5 Mortality Rate (U5MR) in Bangladesh”. Please put citations for this.

**Reply:** We have added citations accordingly or removed the section if it seemed less important.

**Reviewer #2:** Clearly add the specific research gap the present study is addressing.

**Reply:** We have newly added “So far, there have been very few research studies on the SDG 3 indicator variables in Bangladesh. None of these studies have included all of the indicators together, and most of them have used classical time series analysis for forecasting. Our study aims to fill this research gap by forecasting all of the SDG 3 indicators of Bangladesh together using both classical time series analysis and machine learning methods. We will compare the accuracy of the different models and compare the forecasted values with the given target values for the year 2030, which marks the end of the Sustainable Development Goal era.” as the research gap in the Introduction section.

**Reviewer #2:** Material and method section

Material and method section is poor. There are no details of the material included in this section. Additionally, include details about how the data was prepared and processed before being fed into these models. While the descriptions of ARIMA, BRNN, and ENET models are informative, expanding on how these models were specifically applied to your data sets would enhance understanding. Also discuss the metrics used to evaluate the performance of these models.

**Reply:** We have newly added the Cross-validation part, explanation of the methods, model performance metrics, and included details about how the data was prepared and processed before being fed into these models. We have newly discussed how these models were specifically applied to your data sets would enhance understanding. We added lines 143-144 as “Overall, these models were selected because of their relevance and ability to handle the specific challenges posed by small sample sizes or limited data points.”

**Reviewer #2:** Result

Explain why machine learning methods were exclusively used for MMR and Death Rate Due to RTI instead of ARIMA models. This clarification will help in understanding the methodology's adaptability to different datasets.

**Reply:** We added lines 219-223 as “In the context of autoregressive integrated moving average (ARIMA) models, a general guideline suggests that there should be a minimum of 50 observations, preferably exceeding 100, for effective model performance (Box and Tiao 1975). Since there are just 21 and 20 observations of MMR and Death Rate Due to RTI respectively, ARIMA models for those datasets are not ready to be fitted. The analyses were conducted with applying only ML methods for MMR and Death Rate Due to RTI.”

**Reviewer #2:** How it was stated that the series was stationary at first or second difference. The actual test results or statistics that led to this conclusion are not presented. There is no correlogram present in the manuscript, but is mentioned in the text. While writing R2, show 2 as a superscript.

**Reply:** We have newly presented the actual test results or statistics that led to this conclusion of stationary at first or second difference within lines “182-186” and “202-205”. And we have added the correlograms as Fig 2 and Fig 3. While writing R2, we have shown 2 as a superscript.

**Reviewer #2:** In the text when mentioning the table, mention the table number.

**Reply:** We have solved the issue accordingly.

**Reviewer #2:** Mention the value of matrices at both calibration and validation part.

**Reply:** In our study, we conducted a validation process with a tune length of 1000 and a training percentage 75%. However, due to the small size of our dataset, it was not possible to perform a calibration process based on the criteria of model fitting.

**Reviewer #2:** The discussion section is missing.
**Reply:** We have newly added the discussion section.

Thank you sir, for your insightful feedback.

We've incorporated all feedback from the editor and reviewers, ensuring the revised manuscript aligns with PLOS ONE's high standards. We do believe the changes substantially enhance the research quality and contribution.

We reiterate our deep appreciation for their invaluable expertise and insights that greatly improved our work. We trust this revised version merits publication in PLOS ONE.

Thank you for your time and consideration.

Sincerely,

On behalf of all the authors

Md. Maeen Molla (E-mail: [s1710624163@ru.ac.bd](mailto:s1710624163@ru.ac.bd))

M.Phil., Department of Statistics, University of Rajshahi

Rajshahi, Bangladesh.
