# Supplementary material for: Exploring the achievements and forecasting of SDG 3 using machine learning algorithms: Bangladesh perspective
Source: PLoS One. 2025 Mar 4;20(3):e0314466. doi: 10.1371/journal.pone.0314466 (PMC11878945; doi:10.1371/journal.pone.0314466)
Supplement: Attachment — Submitted filename: Response_to_Reviewers_auresp_2.docx [file pone.0314466.s004.docx]

Md. Maeen Molla

M.Phil. Fellow

Department of Statistics

University of Rajshahi

Rajshahi, Bangladesh-6205

07 August 2024

PLOS ONE

Rebuttal Letter for Manuscript [PONE-D-24-00392 and EMID:5f662cd9eff2f8bc]

Dear sir,

We would like to extend our heartfelt appreciation to the academic editor and reviewers for generously devoting their time and offering insightful feedback on our manuscript titled "Exploring the achievements and forecasting of SDG 3 using machine learning algorithms: Bangladesh perspective." We are grateful for the constructive comments provided, as they have enabled us to enhance the overall quality and coherence of our research. In light of the raised concerns, we have meticulously addressed each point in a comprehensive manner as outlined below:

**Reviewer #2:** The current description in the Material and Methods section still appears insufficiently detailed, particularly regarding data sourcing and model specifics. The section lacks essential information, such as: data source, sample size and model details. More comprehensive details are needed about the models employed in the study. Please check some published articles of good journals, to know how to write these.

**Reply:** We have added the data source part as ‘Data availability’ in the lines 131-138 and model specifics in the lines 114-119 as “ARIMA, BRNN, and ENET were chosen for this study because they are well-suited for handling small sample sizes or limited data points. ARIMA is specifically designed for forecasting equally spaced univariate time series data. BRNN, with its bidirectional structure, can effectively utilize information from both past and future states, which can be advantageous when working with a small sample size. ENET, as a regularization technique, can handle the multicollinearity problem and perform feature selection simultaneously, making it a good choice for models with limited data points.” Sample size was mentioned in the lines 70-71, 77-78, 85-86 and 90-91. ARIMA model was not written in details so we have improved the section in the lines 140-146.

**Reviewer #2:** We can only decide a model is generalized or not based on the test data statistics. As it is mentioned that 25% of the data is used for validation set. Please provides the results of the error matrices, based on these values.

**Reply:** We have mentioned the results of the error matrices, based on the validation part for each data set in the Table 1, Table 3 and Table 6.

**Reviewer #2:** Give the citation of the statement: These models, ARIMA, BRNN, and ENET, were chosen because they are well-suited for handling small sample sizes or limited data points.

**Reply:** We have added the citation to the statement.

**Reviewer #2:** Calculating RMAE isn’t standard practice mainly because the square root doesn’t provide additional insights or advantages over MAE in the context of understanding model performance. Additionally, taking the square root of an average of absolute values doesn’t conform to a meaningful statistical measurement.

**Reply:** We understand your concerns regarding the use of the RMAE compared to the MAE. While MAE is indeed a widely accepted metric for model performance evaluation due to its straightforward interpretation, we included RMAE in our analysis for several reasons. RMAE ensures consistency with other metrics like RMSE, allowing for a more direct comparison of the scales of errors, particularly when discussing error metrics with audiences familiar with RMSE. Although RMAE's sensitivity to larger errors is not as pronounced as RMSE, it still highlights scenarios where larger deviations are critical and require attention. We found empirically that RMAE provided additional insights, especially when error distributions had certain characteristics, justifying its inclusion.

**Reviewer #2:** No need to write the details of error matrices in point numbers. Write those in a paragraph style. Please check some published articles of good journals.

**Reply:** We have written the details of error matrices in paragraph style.

**Reviewer #2:** The discussion provides a comprehensive analysis of SDG 3 in the context of Bangladesh and offers valuable insights into the country’s progress and challenges. However, the section lacks a comparative analysis with previous studies. Comparisons to prior research would strengthen the manuscript.

**Reply:** We appreciate your recognition of the comprehensive analysis provided in the discussion section regarding SDG 3 in the context of Bangladesh. We would like to address your suggestion about including a comparative analysis with previous studies. Our work represents a pioneering effort in this specific area, as there are currently no prior studies available that offer a direct comparison with our findings. This research is novel in its approach and scope, focusing on aspects of SDG 3 in Bangladesh that have not been previously explored in the literature. We acknowledge the importance of comparative analysis and will continue to monitor emerging research in this area.

**Reviewer #2:** Please add the funding details data availability and conflict of interest statements.

**Reply:** We have no funding, we added data availability statement and we have no conflict of interest. Moreover, we have selected the answers of the questions related to those statement accordingly while submitting our manuscript.

We've incorporated all feedback from the editor and reviewers, ensuring the revised manuscript aligns with PLOS ONE's high standards. We do believe the changes substantially enhance the research quality and contribution. Additionally, we are interested to publish the whole reviewing process too.

We reiterate our deep appreciation for their invaluable expertise and insights that greatly improved our work. We trust this revised version merits publication in PLOS ONE.

Thank you for your time and consideration.

Sincerely,

On behalf of all the authors

Md. Maeen Molla (E-mail: [s1710624163@ru.ac.bd](mailto:s1710624163@ru.ac.bd))

M.Phil., Department of Statistics, University of Rajshahi

Rajshahi, Bangladesh.
