# Supplementary material for: Exploring the achievements and forecasting of SDG 3 using machine learning algorithms: Bangladesh perspective
Source: PLoS One. 2025 Mar 4;20(3):e0314466. doi: 10.1371/journal.pone.0314466 (PMC11878945; doi:10.1371/journal.pone.0314466)
Supplement: Attachment — Submitted filename: Response_to_Reviewers_auresp_3.docx [file pone.0314466.s005.docx]

Md. Maeen Molla

Lecturer

Department of Statistics

Bangabandhu Sheikh Mujibur Rahman Science and Technology University, Pirojpur

25 September 2024

PLOS ONE

**Rebuttal Letter for Manuscript [PONE-D-24-00392 and EMID:5f662cd9eff2f8bc]**

Dear sir,

We would like to extend our heartfelt appreciation to the academic editor and reviewers for generously devoting their time and offering insightful feedback on our manuscript titled "Exploring the achievements and forecasting of SDG 3 using machine learning algorithms: Bangladesh perspective." We are grateful for the constructive comments provided, as they have enabled us to enhance the overall quality and coherence of our research. In light of the raised concerns, we have meticulously addressed each point in a comprehensive manner as outlined below:

We have thoroughly reviewed our reference list and can confirm that all citations are accurate and current. There are no retracted papers included in our references and we have not identified any need to replace or modify any entries.

**Additional Editor:** Remove the following subheadings under

Materials and methods section: Conceptual framework

Results section: Neuron, Lag, Model adaptation, Temporal consideration, Model complexity

Alpha (fraction), Lambda, Lag, Consideration for alpha (fraction) parameter, Consideration for lambda and regularization

The paragraph lengths should be equal as far as possible.

**Reply:** We have removed the mentioned subheadings and tried to set the paragraph lengths equal as far as possible.

**Additional Editor:** Merge the following headings into one

“ARIMA model fitting for NMR” and “ARIMA model fitting for U5MR” as “ARIMA model fitting”

**Reply:** We have merged the headings.

**Additional Editor:** Make the changes in the following headings

“BRNN parameters” to “BRNN”

“ENET parameters” to “ENET”

**Reply:** We have made the changes.

**Additional Editor:** Improve the quality of the figures (at least 300 dpi)

**Reply:** We have improved the quality of the figures as 300 dpi.

We've incorporated all feedback from the editor, ensuring the revised manuscript aligns with PLOS ONE's high standards. We do believe the changes substantially enhance the research quality and contribution. Additionally, we are interested to publish the whole reviewing process too.

We reiterate our deep appreciation for their invaluable expertise and insights that greatly improved our work. We trust this revised version merits publication in PLOS ONE.

Thank you for your time and consideration.

Sincerely,

On behalf of all the authors

Md. Maeen Molla (E-mail: [s1710624163@ru.ac.bd](mailto:s1710624163@ru.ac.bd))

Lecturer

Department of Statistics

Bangabandhu Sheikh Mujibur Rahman Science and Technology University, Pirojpur

Pirojpur-8500, Bangladesh
